# Henry Thomas Rudge

**Published:** 1903-06

**Authors:** 


### HENRY THOMAS RUDGE.

Henry Thomas Rudge died very suddenly in Pembrokeshire, where he had gone for a much-needed rest and change, on April 10th, of angina pectoris. He was the son of the late C. T. Rudge, a member of an old Gloucestershire family. After some years of work in the firm of Messrs. F. F. Fox & Co, in 1879 he entered as a student at the Bristol Medical School and the Bristol General Hospital. In 1883 he took the diplomas of M.R.C.S. and L.R.C.P. Edin., and then for a short period became Physician's Assistant at Bristol General Hospital.

An opportunity having presented itself, he commenced private practice in 1884 in Redcliff. His skill and resourcefulness and conscientious attention, together with a happy and sympathetic nature, soon gathered round him a large number of patients. By them he was immensely beloved and trusted. It is not too much to say that he was highly respected by all who ever came in contact with him in whatever capacity they did so. The genuine and unaffected outburst of sorrow in Redcliff and elsewhere among all classes was an eloquent testimony to his popularity.

To a very bright and cheery disposition he united intellectual power of a high order, and a zeal and devotion to work which his friends now feel must have told upon his constitution. He was always extraordinarily kind and considerate, especially to the poor and aged, and his delightful gift of sympathy was greatly valued by his patients and friends. He was a loyal Churchman, and as such has successively filled the offices of sidesman, churchwarden, and vestryman of the well-known church of St. Mary Redcliff.

The esteem in which he was held by his brother Freemasons was witnessed to by their presence at his funeral. Indeed, the large number of relatives, friends, and patients, of clergy, members of his own profession, and fellow-parishioners, who assembled at Redcliff Church, was a touching testimony of the place which Henry Rudge occupied in the thoughts and affections of those among whom he had lived and worked. His merry laugh, his bright presence, his thoughtful care, his wise treatment, and his constant, unaffected sympathy will be sadly missed by very many, not only of the patients who fell to his care, but of his medical friends who had the privilege of seeing something of his methodical devotion to work. He died at the early age of 51.

